# DICER governs characteristics of glioma stem cells and the resulting tumors in xenograft mouse models of glioblastoma

**DOI:** 10.18632/oncotarget.10570

**Published:** 2016-07-13

**Authors:** Sheila Mansouri, Sanjay Singh, Amir Alamsahebpour, Kelly Burrell, Mira Li, Merve Karabork, Can Ekinci, Elizabeth Koch, Ihsan Solaroglu, Jeffery T. Chang, Bradly Wouters, Kenneth Aldape, Gelareh Zadeh

**Affiliations:** ^1^ Princess Margaret Cancer Centre and MacFeeters-Hamilton Centre for Neuro-Oncology Research, Toronto, ON, Canada; ^2^ School of Medicine, Koç University, Rumelifeneri Yolu, Sariyer, Istanbul, Turkey; ^3^ Loma Linda University, School of Medicine, Loma Linda, CA, USA; ^4^ Department of Integrative Biology and Pharmacology, McGovern Medical School, University of Texas, Houston, TX, USA; ^5^ Ontario Cancer Institute and Campbell Family Institute for Cancer Research, Princess Margaret Cancer Centre, Toronto, ON, Canada; ^6^ Radiation Medicine Program, Princess Margaret Cancer Centre, Toronto, ON, Canada; ^7^ Department of Medical Biophysics, University of Toronto, Toronto, ON, Canada; ^8^ Department of Neurosurgery, Toronto Western Hospital, University Health Network, 4W-436, Toronto, ON, Canada

**Keywords:** GSC, glioblastoma (GB), DICER, miRNA, radiation resistance

## Abstract

The RNAse III endonuclease DICER is a key regulator of microRNA (miRNA) biogenesis and is frequently decreased in a variety of malignancies. We characterized the role of DICER in glioblastoma (GB), specifically demonstrating its effects on the ability of glioma stem-like cells (GSCs) to form tumors in a mouse model of GB. DICER silencing in GSCs reduced their stem cell characteristics, while tumors arising from these cells were more aggressive, larger in volume, and displayed a higher proliferation index and lineage differentiation. The resulting tumors, however, were more sensitive to radiation treatment. Our results demonstrate that DICER silencing enhances the tumorigenic potential of GSCs, providing a platform for analysis of specific relevant miRNAs and development of potentially novel therapies against GB.

## INTRODUCTION

Although miRNAs play a role in tumor development through their function as both tumor suppressors and oncogenes [1, 2], widespread down-regulation of miRNAs is thought to promote cellular transformation and is observed in several human cancers [1, 3, 4]. Similarly, altered expression of the components of the miRNA processing machinery such as DICER, Exportin 5 (XPO5), and TRBP2 leads to perturbed miRNA expression and may be associated with tumorigenesis [5–10]. DICER is a ribonuclease (RNase) III enzyme that is required for cleavage of pre-miRNAs into their mature 19-23 nucleotide functional form [11]. Therefore, defects in DICER are possible mechanisms for global down-regulation of mature miRNAs. Furthermore, mutations in DICER have been detected in pleuropulmonary blastoma and Sertoli-Leydig cell tumors, among other malignancies [12, 13]. Several studies using mouse models have reported that loss of a single Dicer1 allele leads to oncogenesis in mouse models of lung cancer, retinoblastoma, and lymphoma [14–16]. These findings, along with studies demonstrating that DICER knockdown is associated with global loss of mature miRNAs *in vitro* and *in vivo*, suggest that DICER is functionally linked to oncogenesis [17].

GB is the most malignant primary adult brain tumor and displays a poor clinical outcome as the median survival of patients with the current standard of care remains 12–18 months after recurrence [18]. Most malignant cancers including GB are composed of a heterogeneous population of stem-like cancer cells in which specific miRNAs are downregulated [19, 20]. Glioma stem-like cells (GSCs) can form intracranial tumors that histopathologically resemble GB in orthotopic mouse models and are capable of self-renewal and indefinite proliferation *in vitro* [21]. GSCs are known to display both chemo- and radiation-resistance [22, 23] and reside within specific perivascular niches and around hypoxic centers of the tumors [24–26]. Thus, it is thought that GB pathogenesis and heterogeneity can be orchestrated by GSCs, which can also result in treatment failure [27–30].

Although DICER and global miRNA levels are downregulated in gliomas, complete loss of *DICER1* and miRNA expression have not been reported [14], suggesting that DICER and miRNAs play a critical role in the development of these tumors [31]. In addition, several reports have suggested that DICER knockdown sensitizes human cells to DNA double stranded breaks by demonstrating that DICER is required for ATM-dependent DNA damage response [32] and efficient homologous recombination repair [33]. In this study, we investigated the role of DICER in regulating GSC self-renewal, as well as characterizing tumors generated from these cells in mice as a result of DICER knockdown and their sensitivity to radiation therapy.

## RESULTS

### RISC immunoprecipitation and identification of functional miRNA pool in GSCs

**Figure 1:**
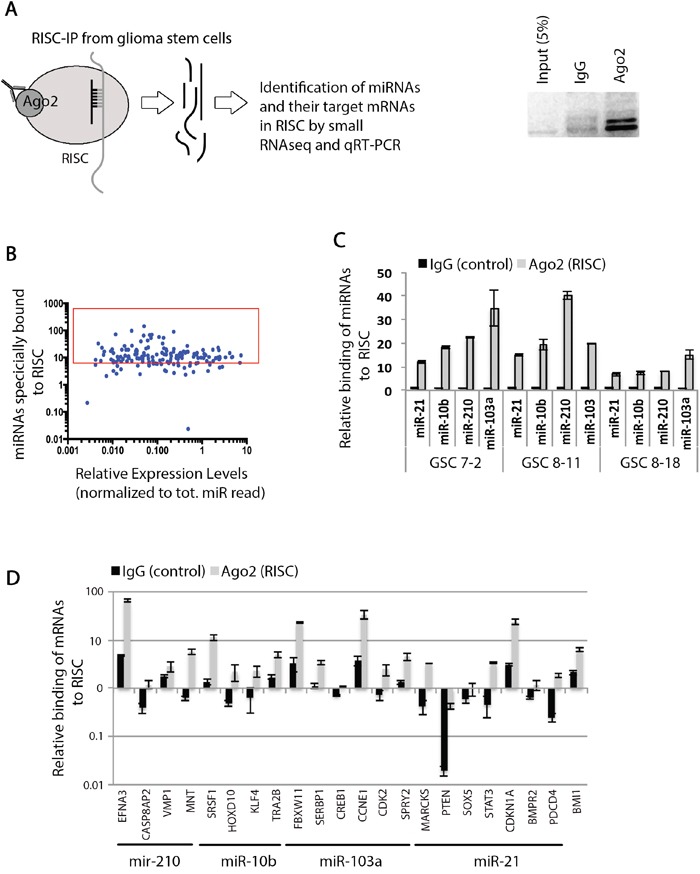
Identification of functional miRNAs and their target mRNAs in RISC in GSCs **A.** A schematic demonstrating the principles of RISC-IP experiment and analysis of bound miRNAs and their target mRNA molecules. **B.** Graph shows that approximately 150 mature miRNAs are specifically bound to RISC complex when normalized to the negative control IgG sample. **C.** qRT-PCR validation of specific miRNAs bounds to RISC in GSC 7-2, 8-11, and 8-18 cells. We used −2^ΔΔCt^ method to estimate the relative abundance of miRNAs relative to IgG negative control. **D.** Specific target mRNAs were evaluated for their abundance in RISC complex using qRT-PCR after normalization to negative control IgG. The −2^ΔΔCt^ method was used to estimate the abundance of mRNAs relative to IgG sample. Graph shows data +/−SD from two independent experiments performed in triplicate.

In order to identify potentially functional miRNAs in GSCs, we immunoprecipitated the RISC complex, along with its associated miRNAs and mRNAs from GSC 7-2 cells (Figure 1a). We used an antibody specific to AGO2 protein, which is the main enzymatic component of the mammalian RISC. Using small RNA sequencing, we found a total of 150 miRNAs that were specifically bound to beads (>3-fold) in the RISC complex compared to the control IP using non-specific IgG raised in a host where AGO2 antibody was raised (Figure 1b and Supplementary Table S3). We validated four of the miRNAs for their incorporation into the RISC complex by qRT-PCR; namely, miR-103a, miR-210, miR-10b, and miR-21 (Figure 1c). We chose these miRNAs because of their established role in regulating pluripotency, cell cycle, and proliferation [44–47]. We also analyzed the presence of specific mRNAs that are targets for these miRNAs within the RISC complex with qRT-PCR analysis. We specifically investigated mRNAs that code for genes regulating pluripotency and cell cycle, such as *SOX2*, *BMI1*, *STAT3*, *CDKN1A*, and *CCNE1*. Our results indicate that these mRNAs are associated with the RISC complex in GSCs (Figure 1d).

### DICER knockdown alters GSC characteristics

**Figure 2:**
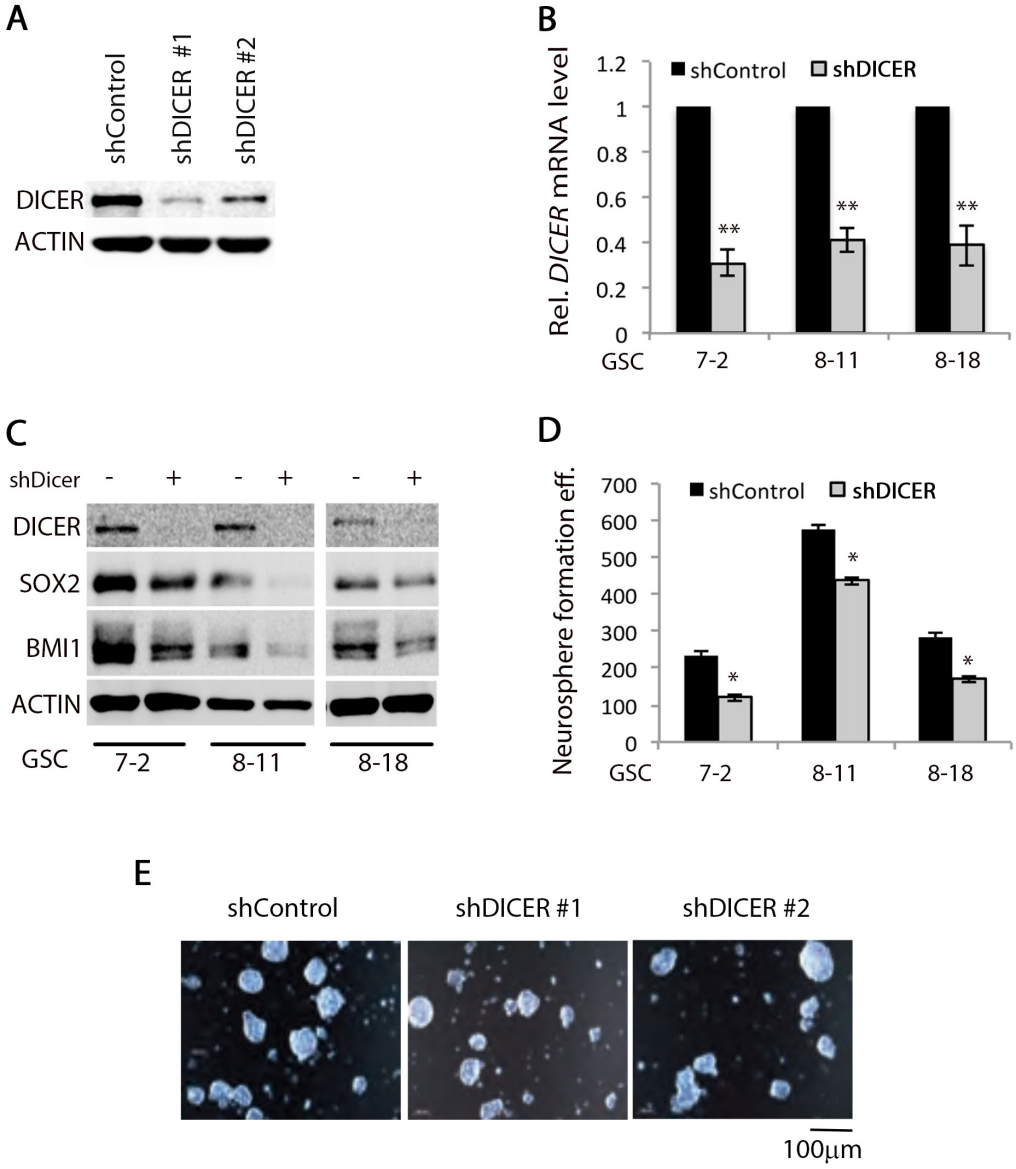
DICER1 knockdown alters GSC characteristics Two different shRNAs targeting *DICER1* mRNA were transduced into GSCs using lentiviral constructs. Upon stable transfection and selection with puromycin, **A.** Western blot analysis of GSC lysates was performed using DICER1-specific antibody to show that both constructs significantly decreased DICER protein level. ACTIN was used as internal control. **B.** qRT-PCR analysis demonstrated that shDICER constructs decreased *DICER1* mRNA level in all three GSC lines. We used −2^ΔΔCt^ method to calculate relative abundance of *DICER1* mRNA in each sample. We used *GAPDH* as internal control mRNA for normalization and the data presented are mean +/−SD from two independent experiments each performed in triplicate. * denotes *p < 0.05* and ** denotes *p < 0.01*. **C.** Analysis of DICER knockdown effect on expression of stem cell marker genes was performed by Western blot assay using antibodies specific for SOX2 and BMI1 in GSC 7-2, 8-11, and 8-18 lines. ACTIN was used as internal control. **D.** Single cell GSC cultures were grown in GSC basal media containing growth factors at a seeding density of 1,000 cells per well in 6-well plates. The resulting spheres (100-150 μm) were counted 14 days later and graphs present the number of spheres formed by shControl or shDICER GSC 7-2, 8-11, and 8-18 cells. Data presented are +/−SD from two independent experiments each performed in triplicate. * denotes *p* < 0.05. **E.** Representative images were taken using a light confocal microscope to illustrate the size range and appearance of GSC 7-2 spheres 14 days after plating as single cells in GSC basal media.

We first evaluated expression of the components of the miRNA processing machinery in GSCs in comparison to the human neural stem cell line (hNSC D341) by Western blot analysis. DICER expression was lower in all three GSC lines compared to hNSC D341 cells; however, no difference was detected in AGO2 expression (Supplementary Figure S1c). We then silenced DICER expression in three different GSC lines (GSC 7-2, 8-11, and 8-18) using two independent shRNA constructs targeting *DICER1* mRNA. Decrease in DICER protein and mRNA levels were confirmed by Western blot and qRT-PCR, respectively. DICER protein and mRNA levels decreased using both shRNA constructs, while shDICER #1 resulted in greater knockdown in DICER expression (Figure 2a, 2b, and 2c). We then analyzed the effect of DICER knockdown on expression of stem cell marker genes SOX2 and BMI. Upon DICER silencing, SOX2 and BMI1 decreased in all three GSC lines tested (Figure 2c), suggesting that these cells may have decreased ability to self-renew and form neurospheres. We then tested the neurosphere formation efficiency of DICER knockdown GSCs. Equal numbers of cells (1,000 cells/well) from each condition were seeded into triplicate wells in 6-well plates. The resulting spheres—ranging in diameter from 100-150μm—were counted 14 days later. DICER silencing reduced neurosphere formation efficiency by decreasing the number and sizes of neurospheres (Figure 2d and 2e). This is in line with other reports demonstrating a correlation between the expression of stem cell marker gene *SOX2* and the ability of GSCs to self-renew [48–50].

### Effect of DICER knockdown on cellular miRNA biogenesis

Since DICER is the key enzyme required for processing of precursor miRNAs (pre-miRNAs) into mature miRNAs in the cytoplasm, it is likely that DICER silencing would decrease global cellular miRNA biogenesis. To confirm this, we performed a miRNA PCR array analysis on total small RNAs isolated from shDICER and shControl GSC 7-2 cells. Approximately 170 miRNAs were expressed above background in GSCs and the majority of these are known to be highly abundant in these cells [51]. A total of 52 miRNAs decreased greater than 2-fold upon DICER knockdown and no increase was detected in any of the miRNAs (Table 1 and Supplementary Table S4). This was expected as DICER knockdown leads to widespread down-regulation of mature miRNAs. The majority of the decreased miRNAs are known tumor suppressors, such as members of the let-7 family of miRNAs [14, 15, 52]. On the other hand, oncogenic miRNAs such as miR-21 decreased as well. It is, however, not clear whether global downregulation of miRNAs enhances the ability of GSCs to form tumors or whether the loss of specific tumor suppressor or oncogenic miRNAs would increase tumorigenicity of GSCs. Therefore, this is an important avenue of future investigation.

**Table 1:** List of miRNAs decreased (>3-fold) in *DICER* knockdown versus control GSC 7-2 cells based on miRNA PCR array results

miRNA	Fold Change
hsa-let-7b-5p	−18.1
hsa-miR-98-5p	−14.0
hsa-let-7i-5p	−11.8
hsa-let-7d-5p	−9.2
hsa-let-7g-5p	−7.3
hsa-miR-301a-3p	−6.2
hsa-miR-129-2-3p	−5.4
hsa-let-7f-5p	−5.4
hsa-miR-99b-5p	−4.8
hsa-let-7c	−4.5
hsa-miR-652-3p	−4.3
hsa-miR-107	−4.2
hsa-miR-210	−4.0
hsa-miR-330-3p	−3.9
hsa-miR-105-5p	−3.8
hsa-miR-342-5p	−3.7
hsa-let-7e-5p	−3.6
hsa-miR-103a-3p	−3.5
hsa-miR-345-5p	−3.5
hsa-miR-484	−3.5
hsa-miR-181c-5p	−3.4
hsa-miR-129-1-3p	−3.3
hsa-miR-93-5p	−3.1
hsa-miR-200c-3p	−3.1
hsa-miR-421	−3.0
hsa-miR-146b-5p	−3.0
hsa-miR-324-5p	−3.0
hsa-miR-542-3p	−3.0

**Figure 3:**
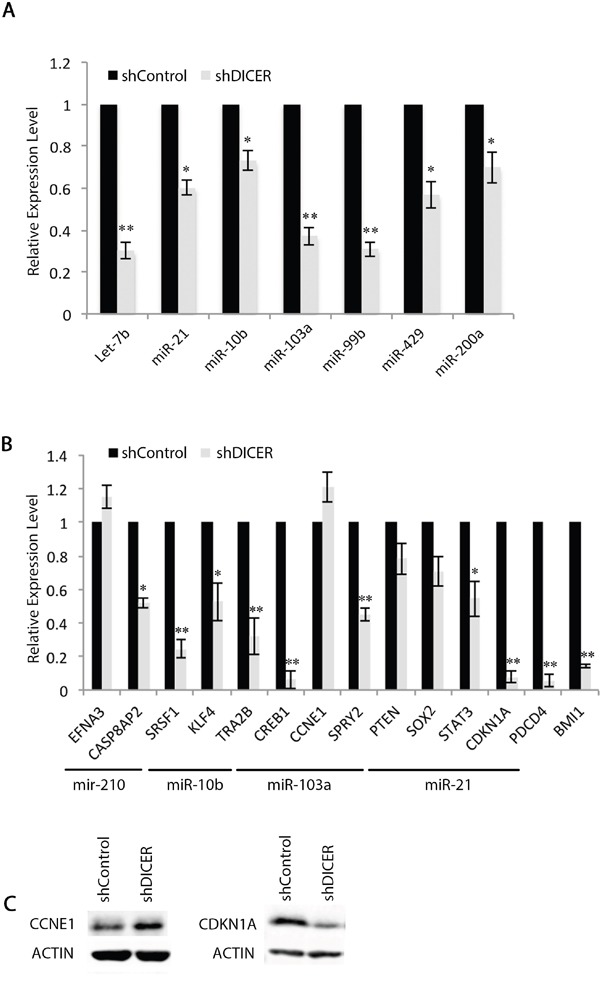
Expression of specific mRNAs do not correlate with their targeting miRNA expression profiles in GSCs **A.** qRT-PCR validation of changes in specific miRNAs in shDICER GSC 7-2 compared to shControl cells. We used −2^ΔΔCt^ method to estimate the relative abundance of miRNAs normalized to that of *RNU6*. **B.** qRT-PCR analysis was performed to determine relative expression of target mRNAs in shDICER relative to shControl GSC 7-2. The −2^ΔΔCt^ method was used to determine relative abundance of the indicated mRNAs normalized to that of *GAPDH* or *ACTB* mRNAs. Graph shows data +/−SD from two independent experiments performed in triplicate. * denotes *p < 0.05* and ** denotes *p < 0.01*. **C.** Western blot analysis was performed using antibodies specific for CCNE1 (Cyclin E1) and CDKN1A (p21) to determine correlation of protein and mRNA expression for these genes. ACTIN was used as internal control.

We then validated the expression of a select number of miRNAs (let-7b, miR-10b, miR-21, miR-99b, miR-429, and miR-200a) as they target mRNAs that encode for stemness and proliferation markers including SOX2, BMI1, and CCNE1. We determined miR-99b levels as it is known to target *mTOR* mRNA and confer resistance to radiation in human pancreatic cancer cell lines [53]. Our results confirm that DICER silencing lowers the expression of the miRNAs tested (Figure 3a). It is thought that mRNA degradation often occurs subsequent to translational repression by miRNAs [54, 55]. Therefore, we examined the level of mRNA targets for some of the miRNAs that decreased with DICER silencing. The majority of mRNAs tested—including *SOX2*, *STAT3*, and *CDKN1A* (*p21*)—were lower in shDICER compared to shControl cells, with the exception of *Cyclin E1* (*CCNE1*) and *EFNA3* (Figure 3b). Expression of Cyclin E1 increased at the protein level, which correlates with decreased expression of its targeting miRNA (miR-103a; Figure 3c; [44]). Lower expression of *SOX2*, *BMI1* and *CDKN1A* mRNAs in DICER knockdown cells is in agreement with their decreased protein expression and also correlates with the reduced ability of GSCs to form neurospheres. This is, however, contrary to what is expected as the level of their targeting miRNAs decreased as well.

### DICER knockdown in GSCs results in formation of more aggressive tumors in mice

**Figure 4:**
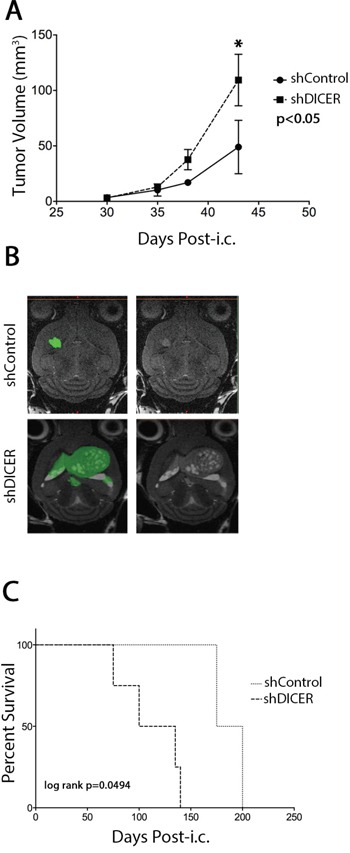
Effect of DICER1 knockdown on tumors generated from GSC 7-2 cells in mice **A.** Volumetric analysis of tumors on days 30, 35, 38, and 43 post-intracranial (i.c.) injections. MRI analyses of tumor volumes were performed on three mice in each group and mean values +/−SD is presented. * denotes *p* < 0.05. **B.** Representative images of brain sections of intracranial tumor-bearing mice in each treatment group, with and without color overlay, to demonstrate tumor boundaries. **C.** Kaplan-Meier survival analysis of mice implanted with shControl or shDICER GSC 7-2 cells (1,000 cells/mouse). Mice were monitored for any neurological deficits and moribund mice were sacrificed.

In order to use GSCs as tumor-initiating cells in our experiments, we first performed intracranial injection of GSC lines (GSC 7-2, 8-11, and 8-18) to determine their ability to form GB-like tumors. Indeed these cells developed tumors in mouse xenografts, that resemble patient GB, showing features such as hypoxic centers and necrosis, but displayed limited heterogeneity compared to patient tumors (Supplementary Figure S1). Therefore, it is possible that these GSCs are in a progenitor state with a more limited self-renewal capacity than actual stem cells. To test the effect of DICER knockdown on the tumorigenic potential of GSCs, GSC 7-2 and 8-11 cells stably expressing shDICER or shControl constructs were intracranially injected into the right frontal cortex of NOD/SCID mice. The mice were monitored for tumor growth and volumetric analysis was performed over time using MRI. DICER knockdown GSCs generated larger tumors relative to shControl GSCs (2.8-fold larger on day 43 post-intracranial injection (post-i.c.; p<0.05; Figures 4a and 4b). Consistently, Kaplan-Meier survival analysis showed that shDICER GSCs lead to significantly shorter survival in mice compared to shControl GSCs (median survival 101 vs. 207 days; log-rank p = 0.0494; Figure 4c).

### Molecular characterization of DICER knockdown tumors

**Figure 5:**
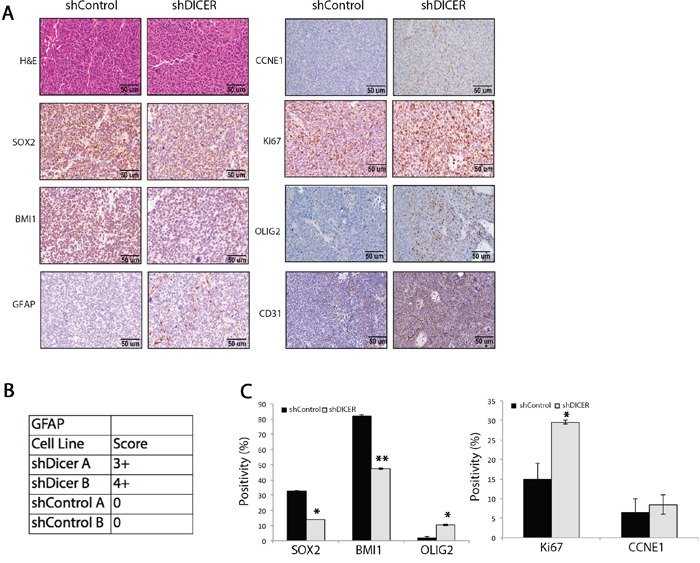
Immunohistochemical analysis of stemness, proliferation, and differentiation marker genes in shDICER and shControl tumors **A.** Immunohistochemical (IHC) analysis of FFPE brain sections collected from mice in each group was performed using the indicated antibodies. **B.** The table demonstrates GFAP positivity score and **C.** the graphs represent quantitative analysis of cells that stained positive for the indicated marker genes from multiple fields of view. * denotes *p* < 0.05 and ** *p* < 0.01.

We then evaluated the expression of stem cell and prodifferentiation marker genes including *SOX2* (stem marker), *BMI1* (stem marker), *GFAP* (astrocytic marker), and *OLIG2* (oligodendrocytic marker) in tumors arising from shDICER and shControl GSCs by immunohistochemistry (IHC). Consistent with our *in vitro* observations, shDICER tumors showed a statistically significant decrease in expression of SOX2 and BMI1, while the expression of GFAP and OLIG2 significantly increased (Figures 5a, 5b, 5c). On the other hand, expression of proliferation marker genes, *MKI67* (*MIB-1*) and *Cyclin E1* (*CCNE1*), was higher in shDICER tumors, which is consistent with larger volume of DICER knockdown tumors. Similar results were obtained through IHC analysis of the same marker genes in tumors developed from intracranial injection of GSC 8-11 cells (Supplementary Figure S2). Additionally, we confirmed the specificity of our antibodies by performing IHC analysis on tumor tissue sections using secondary antibodies alone (Supplementary Figure S3).

**Figure 6:**
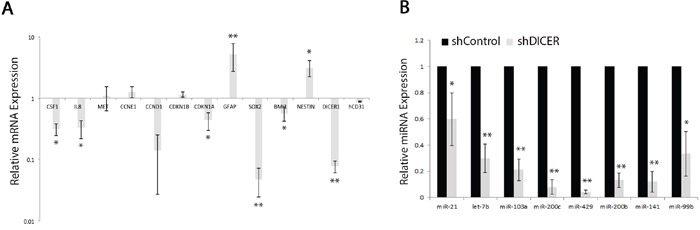
Analysis of specific miRNA and mRNA expression profiles in shDICER and shControl tumors **A.** Total RNA (1 μg) isolated from tumors collected from xenografts mice, was reverse transcribed, and subjected to qRT-PCR analysis using gene specific primers. The −2^ΔΔCt^ method was used to determine relative abundance of the indicated mRNAs normalized to that of *GAPDH* or *ACTB* mRNAs. **B.** Total cellular RNA was also assessed for expression of the indicated mature miRNAs using Taqman miRNA assay kits. The −2^ΔΔCt^ method was used to estimate the relative abundance of miRNAs normalized to that of *RNU6*. The data presented are mean values +/−SD for expression analysis performed on two independent tumors collected from each group and were subjected to qRT-PCR analysis in technical triplicates. * denotes *p* < 0.05 and ** *p* < 0.01.

We then performed qRT-PCR on RNA isolated from shControl and shDICER tumors to evaluate relative abundance of several mRNAs coding for cell cycle regulators, stemness, and prodifferentiation markers including *SOX2*, *BMI1*, *CCNE1*, *GFAP*, and *NESTIN* (Figure 6a), as well as their corresponding targeting miRNAs, let-7b, miR-21, miR-10b, miR-103a, and miR-99b (Figure 6b). Consistent with our *in vitro* results, shDICER tumors expressed lower level of let-7b, miR-99b, miR-103a, and miR-200 miRNA family members (miR-429, miR-200c, miR-141 and miR-200b) compared to shControl tumors. Furthermore, the level of some of their target mRNAs such as *SOX2*, *BMI1*, *CDKN1A*, *and STAT3* also decreased upon DICER silencing. On the other hand, expression of *GFAP* and *NESTIN* mRNAs increased, which may be due to increased transcription and/or stability of these mRNAs. Similar to our *in vitro* data, no change was detected in the level of *CCNE1* and *CD31* mRNAs, suggesting that increased expression of these genes at the protein level may be due to reduced level of specific miRNAs that target their 3′ UTR, such as miR-103a for *CCNE1* and miR-126 for *CD31* [56].

### DICER-deficient tumors are more sensitive to radiation treatment

**Figure 7:**
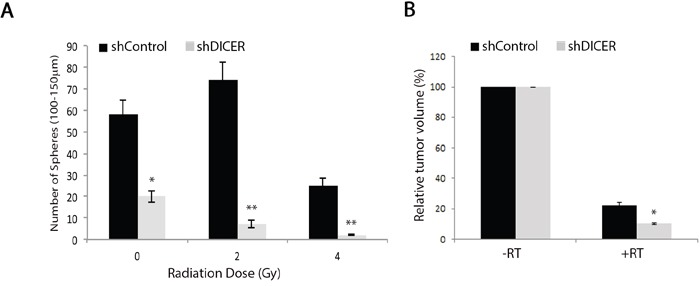
DICER knockdown increases sensitivity of GSCs and their resulting tumors to radiation treatment **A.** GSC 7-2 cells (shDICER or shControl) were treated with one dose (0-4Gy) of ionizing radiation, plated at a density of 1,000 cell/well, and the resulting spheres (100-150 μm in diameter) were counted 14 days later. **B.** Mice injected with shControl and shDICER treated GSC 7-2 cells were treated with a single dose of 6Gy radiation targeted to the right frontal cortex of the brain 14 days post-intracranial (i.c.) injections. MRI volumetric analyses of the resulting tumors were performed on 3 mice in each group 43 days post-intracranial injection and mean values +/−SD are presented. * denotes *p* < 0.05 and ** *p* < 0.01.

Poor response of GB tumors to treatment with ionizing radiation has been attributed to radio-resistance of the small population of GSCs found within these tumors [22, 57]. DICER knockdown has been shown to sensitize human cells to DNA double stranded break by suppressing the ATM-dependent DNA damage response [32] and reducing the efficiency of homologous recombination repair [33]. To test the effect of DICER silencing on the intrinsic radiosensitivity of GSCs and radiation response of the resulting tumors, we exposed GSCs to a single fraction of varying doses of ionizing radiation (0-4Gy), followed by analysis of colony formation efficiency 14 days later. We found that shDICER GSCs generated relatively fewer neurospheres compared to shControl GSCs with all radiation doses tested (Figure 7a). We also tested the sensitivity of tumors arising from shDICER GSCs to radiation by treating mice with one dose (6Gy) of irradiation 14 days following intracranial injection. MRI volumetric analysis showed that irradiated shDICER tumors grew smaller relative to shControl tumors (Figure 7b); specifically, irradiation resulted in 10-fold decrease in volume of shControl tumors, while the volume of shDICER tumors decreased by approximately 15-fold on average.

## DISCUSSION

Several studies have demonstrated that an optimal level of DICER may be necessary to maintain survival, proliferation, and invasiveness of cancer cells in various malignancies [58, 59]. In this study, we investigated the role of DICER in regulating the tumorigenic properties of GSCs and characterized the resulting tumors' proliferative properties and resistance to radiation therapy. DICER knockdown in GSCs resulted in widespread down-regulation of cellular miRNAs, the majority of which were tumor suppressor miRNAs, such as *let-7a* and *let-7b*; however, not all miRNAs were reduced to the same extent and this is likely due to the fact that each step of the general miRNA biogenesis pathway differentially fine-tunes miRNA expression. Recent studies have revealed that not all miRNAs are created equally and different mechanisms allow for the specific regulation of individual miRNAs, resulting in unique expression patterns in different tissues. In addition, different miRNAs play different roles in various tissues and depending on their biological role, certain circumstances may favor expression of specific miRNAs at transcriptional or post-transcriptional stages [60, 61]. DICER knockdown also lowered the neurosphere formation ability of GSCs, suggesting that disruption of the miRNA processing machinery decreases the stemness properties of these cells, likely via decreased expression of specific miRNAs. Consistently, we found that expression of multiple stem cell maker genes including SOX2, SOX5, and BMI1, was reduced *in vitro* and *in vivo* at both protein and mRNA levels. This was contrary to what was expected, as the levels of some of the miRNAs targeting these mRNAs—such as miR-200 family members—were also reduced, suggesting that knockdown of DICER likely inhibits transcription of these mRNAs rather than suppressing their translation via specific miRNAs.

On the other hand, we found increased expression of prodifferentiation marker genes GFAP and OLIG2, the cell cycle regulator CCNE1, endothelial cell marker CD31, and the proliferation marker Ki67 (MIB-1) in shDICER tumors. The level of CDKN1A (p21), however, was reduced at both protein and mRNA levels in shDICER tumors. Therefore, the observed increase in growth of DICER knockdown tumors could be explained by changes in expression of cell cycle regulators CCNE1 and CDKN1A. It is well established that CDKN1A inhibits cell cycle progression through the G1 phase by directly inhibiting the activation of the CCNE1/CDK2 complex [62]. Moreover, a recent study uncovered the role of BMI1 in regulating genome stability through positive regulation of CDKN1A, and thereby, reducing CCNE1 activity in cells [63]. Our results indicate that BMI1 expression is reduced in shDICER GSCs *in vitro* and their corresponding tumors *in vivo*. Furthermore, the overall increase in size of tumors arising from shDICER GSCs was in agreement with reduced overall survival of mice compared to the control group. This finding is consistent with reports correlating lower DICER expression with poor prognosis in other malignancies [64, 65]. Taken together, our data indicate that DICER knockdown may cause a transitional shift of GSCs from a stem cell like-state to a more differentiated and proproliferative progenitor state.

The increase observed in CCNE1 and CD31 proteins is likely due to a post-transcriptional mechanism upon reduction in their targeting miRNAs, including *miR-103a* (targets *CCNE1*; [44]) and *miR-126* (targets *CD31*; [56]), both of which decreased in shDICER relative to shControl GSCs. However, no change was detected in relative abundance of the mRNAs for these genes possibly due to the heterogeneous nature of GB tumors, which may cause masking of regional changes in mRNA expression when the bulk of the tumor is processed. Additional experiments using 3′ UTR reporter constructs—with or without miRNA targeting sites—must be performed to determine the role of these miRNAs in regulating the translation of their specific target mRNAs within the context of GSCs.

It is also interesting to note increased expression of CD31 (a marker for endothelial cells) in shDICER relative to shControl tumors, as shDICER directly affects miRNAs in GSCs which do not contribute directly to blood vessel formation in GB tumors. In fact, the ability of GSC to generate functional endothelial cells is highly controversial [66, 67], but the role of miRNAs in this context has not been examined. DICER silencing may also perturb the level of miRNAs that regulate the expression of angiogenic factors by GSCs, as is the case for members of the miR17-92 cluster in different subsets of cancer cells [68]. Finally, miRNAs may be involved in angiogenesis through their intercellular trafficking in exosomes [69]. While detailed analyses of these possibilities are outside of the scope of this manuscript, this is an important avenue that needs to be explored in the future.

We also found that DICER knockdown increased the sensitivity of GSCs and their resulting tumors to gamma irradiation, as the volume of gamma irradiated shDICER tumors were significantly lower relative to their non-irradiated counterparts, in addition to reduced neurosphere formation efficiency of shDICER GSCs. Wei et al. demonstrated that miR-99b confers resistance to radiation in human pancreatic cancer cell lines by targeting mTOR mRNA [53]. We found that miR-99b was reduced in shDICER GSCs and their resulting tumors in mice. These results indicate a potential mechanism through which DICER down-regulation leads to radiation sensitivity through reduction of miR-99b. Additionally, activation of CCNE1 results in replicative stress and thereby, leads to chromosomal instability [70], which may account for increased radiation sensitivity upon DICER knockdown. Cumulatively, our data suggest that DICER plays a pleiotropic role in GSCs, modulating the growth properties of glioma tumors arising from these cells and their response to DNA damage-inducing agents, leading to formation of larger tumors that are more sensitive to radiation. Future work will likely shed light on additional mechanisms through which DICER enhances tumorigenesis and modifies therapeutic response in glioma.

## MATERIALS AND METHODS

### Glioblastoma stem cell (GSC) culture

GSCs were derived from freshly resected tumor samples from GB patients at the University of Texas MD Anderson Cancer Center as per guidelines set by institutional review board and were described previously [34]. GSCs were maintained in culture as neurospheres in defined DMEM/F12 media in presence of growth factors EGF (20ng/ml; Cat. No. 01-107; EMD Millipore), recombinant βFGF (20ng/ml; Cat. No. 233-FB-025; R&D systems), and B27 growth supplement containing vitamin A (1:50 working concentration; Cat. No. 12587-010; Life Technologies). Fresh media was added every third day of culture (30% v/v) and the cultures were passaged when average neurosphere size reached approximately 100-150μm in diameter. To assess neurosphere formation ability, GSCs were seeded at a density of 1,000 cells per well in 6-well plates in triplicate and the resulting spheres (100-150 μm in diameter) were counted 10-14 days later.

### RISC immunoprecipitation

GSCs were harvested and treated with Accutase for 3min at 37°C to generate single cell suspension. These single cells were pelleted, washed twice with ice-cold PBS and re-suspended in equal volume of polysome lysis buffer containing RNAse OUT (40 U/mL; Cat. No. 10777-019; Invitrogen) and protease inhibitor cocktail (Cat. No. 04693116001, Roche) and then stored at −80°C. For each sample, approximately 120 μl of 50% Protein G agarose slurry (Cat. No. 16-266; EMD Millipore) was pre-coated with 5 μg of Argonaute 2 (AGO2) antibody or matched IgG control antibody for 3h at 4°C. The frozen cell pellets were quickly thawed and spun at 14,000xg for 10min at 4°C. The supernatant was used in immunoprecipitation reaction at a final volume of 1ml. After quick mixing, 5% of the mix was saved as input, followed by tumbling the reactions end-over-end for 3h at 4°C. At the end of the incubation period, beads were treated with proteinase K for 30min at 55°C. The RNA was then isolated by acid phenol chloroform method and precipitated with 50 μl of 5M ammonium acetate, 15μl of 7.5M lithium chloride, 5μl of 5mg/ml glycogen and 850μl of absolute ethanol.

### Small RNA sequencing of RISC-IP miRNAs

Total small RNA isolated from RISC-IP experiment was subjected to next generation sequencing using an Illumina HiSeq 2000 sequencer and obtained an average of 66 million reads per sample. We verified the quality of the resultant reads using FASTQC (http://www.bioinformatics.babraham.ac.uk/projects/fastqc/). Next, we trimmed the adapter sequences and estimated the read counts using miRExpress [35], using miRBase 19 as a reference [36]. Finally, we normalized the counts to reads per million mapped. MiRNAs were determined to be altered by comparing the number of reads for a particular miRNA to the total number of miRNA reads per library.

### DICER knockdown in GSCs

Two lentiviral short hairpin RNA (shRNA) constructs against DICER1 (shDICER #1 and shDICER #2) were used to knock down DICER in GSCs and a non-specific shRNA construct was used as negative control (shControl) as previously described [37]. Briefly, approximately 0.3 MOI of virus particles were used to transduce GSCs to ensure minimal number of genomic integrations. The transduced cells were selected with puromycin (0.5 μg/ml) for 48h post-transduction and maintained under selection for additional 14 days to generate stable clones. Knockdown efficiency was evaluated by qRT-PCR and Western blot assays.

### Intracranial tumorigenicity assay

Intracranial transplantation of GSCs into NOD/SCID mice was carried out as described previously [38]. Briefly, exponentially growing GSC cultures were treated with Accutase to generate single cell suspension. Viable cells were counted on Vi-Cell XR cell counter (Beckman-Coulter). Cells were kept on ice before injections and approximately 2×10^3^ cells, re-suspended in 6μl PBS, were injected in frontal cortex of 3-Week-old mice. Mice were monitored for any neurological symptoms and moribund mice were sacrificed.

### Radiation therapy

A single fraction of 6 Gray (Gy) radiation was delivered on day 14 following intracranial injection using a cone-beam CT image-guided small animal irradiation system (XRT225Cx, Precision X-Ray, Inc.) in which mice were positioned in an in-house, custom-built stereotactic immobilization device, as previously described [39]. Irradiation of GSCs in culture was performed following Accutase treatment and re-suspension in basal media containing growth factors. Single cells were counted and 1,000 cells were plated in triplicate in 6-well plates. The cells were irradiated with the indicated doses of radiation and resulting neurospheres (100-150μm in diameter) were counted 14 days later.

### Magnetic resonance imaging (MRI) and volumetric analysis

MRI was performed with a 7 Tesla Biospec 70/30 (Bruker Corporation), using the B-GA12 gRTient coil insert and 7.2cm inner diameter, linearly polarized volume resonator coil for radiofrequency transmission, as detailed previously [39]. Mice were monitored for tumor development and growth at various time points post intracranial injection. Volumetric analysis was conducted using Mimics^®^ software (Belgium, Materialise). Mimics^®^ is a software for processing medical images and creating 3D models [40, 41]. Volumes of tumors were calculated by selecting and mapping the lesion areas. For this purpose, upper threshold and degree of contrast were used as the main steps in the process. Threshold values that were best fit for the lesions were selected consistently among tumors. To emphasize the difference between the abnormal mass and healthy tissues, different workflow windows were selected for contrast analysis.

### Preparation of whole cell extract and western blot analysis

GSC pellets were lysed in RIPA lysis buffer containing protease inhibitors (Cat. No. 04693116001; Roche) followed by a 30min incubation on ice. The cell lysates were centrifuged at >15,000xg for 10min to remove cell debris. Protein quantification was performed using Pierce™ BCA Protein Assay Kit (Cat. No. 23225; Thermo Scientific) following manufacturer's instructions. Approximately 25-30μg total cellular protein was separated on a 10% SDS-PAGE. The proteins were then transferred to nitrocellulose membrane, which was blocked with 5% non-fat milk or BSA in TBS with 0.1% Tween 20 (TBS-T). The membranes were incubated overnight with primary antibodies at 4°C, followed by 3 washes with 1x TBS-T. Primary antibodies against DICER1 (Cat. No. 3363; 1:1,000; Cell Signaling), AGO2 (Cat. No. ab32381; 2 μg/ml; Abcam), ACTIN (Cat. No. A5316; 1:5,000; Sigma), SOX2 (Cat. No. 3579; 1:1,000; Cell Signaling), BMI1 (Cat. No. 6964; 1:1,000; Cell Signaling), CDKN1A (Cat. No. sc-397; Santa Cruz), and CCNE1 (Cat. No. Ab3927; Abcam) were used in this study. Either IRDye^®^ 800CW (anti-mouse: Cat. No. 926-32212; anti-rabbit: P/N 926-32213; LI-COR) and/or IRDye 680RD secondary antibodies (anti-mouse: Cat. No. 926-68072; anti-rabbit: Cat. No. 926-68073; LI-COR) were used to scan the membrane using the Odyssey detection system (LI-COR Biosciences).

### Total RNA isolation from mouse tumors

Total RNA was isolated from tumors that were embedded and snap-frozen in OCT using TRIzol Reagent (Cat. No. 15596-026; Invitrogen). Briefly, mice were sacrificed according to institutional guidelines and as previously described. Approximately 30min prior to sacrifice, Evans blue dye (2%; 1ml/kg) was injected intravenously to highlight increasing permeability. Brains were removed from perfused mice, the blue-colored region corresponding to the tumor was excised and then embedded in OCT freezing compound (Tissue-Tek) in a 1cm^2^ mould. The samples were snap frozen on dry ice and stored at −80°C [42]. Tumor purity was determined by Haematoxylin and Eosin (H&E) staining of frozen 5μm tumor sections and examined by a neuropathologist, Dr. K. Aldape. Approximately 25mg of frozen tumor block was used for RNA extraction using TRIzol reagent following manufacturer's instructions.

### Quantitative reverse transcriptase PCR (qRT-PCR) for mRNAs and miRNAs

Total RNA was isolated from GSCs (shControl and shDICER) using TRIzol following manufacturer's instructions. Approximately 1 μg total RNA was converted to cDNA using SuperScript^®^ VILO cDNA Synthesis Kit (Cat. No. 11754250; Thermo Fisher Scientific) following manufacturer's instructions. Gene specific primers (Supplementary Table S1) were used along with Fast SYBR^®^ Green Master Mix (Cat. No. 4385612; Life Technologies) to quantify expression level of specific genes using StepOne Plus™ Real-Time PCR System (Life Technologies). The amount of mRNA in each sample was normalized to the amount of *GAPDH* or *ACTIN* mRNAs and relative abundance of individual mRNAs were determined by −2^ΔΔCt^ method.

Enrichment of miRNAs was accomplished by processing samples through mirVana™ miRNA Isolation Kit (Cat. No. AM1560; Life Technologies) following manufacturer's instructions. Approximately 250ng of miRNA pool was then used for quantification of individual miRNAs by miScript^®^ miRNA PCR Array kit (Cat. No. MIHS-3216ZE-12) and miScript SYBR^®^ Green PCR Kit (Cat. No. 218073; Life Technologies). Array data were analyzed using the software available at http://pcrdataanalysis.sabiosciences.com/mirna. For validation of individual miRNAs, approximately 20ng of total RNA was reverse transcribed using SuperScript III reverse transcriptase (Cat. No. 12574026; Thermo Fisher Scientific) and measured by qRT-PCR using TaqMan miRNA assay kits (Applied Biosystems) according to the manufacturer's instructions. MiRNA amounts were normalized to that of *RNU6* or *RNU44* small nuclear RNAs as internal controls and the −2^ΔΔCt^ method was used to determine the relative abundance of miRNAs.

### Immunohistochemistry and data analysis

Immunohistochemistry was performed on 5μm thick tumor sections using heat-induced antigen retrieval method as described previously [43]. Briefly, slides were de-paraffinized in xylene for 30min followed by gradual hydration in decreasing concentrations of ethanol. Antigen retrieval was achieved by incubation in a sodium citrate buffer (pH 6) and pressure cooked for 20min. Endogenous peroxidase activity was blocked by incubation for 20min in 3% hydrogen (in methanol). Non-specific epitopes were blocked by incubation with 10% normal goat serum or bovine serum albumin. Tissue sections were then incubated with corresponding primary antibodies at 4°C overnight (Supplementary Table S2). The detection system consisted of Dako EnVision System, which contains a peroxidase-conjugated polymer with goat anti-mouse or goat anti-rabbit immunoglobulins (Cat. No. K4001; Dako). Visualization of primary/secondary antibody interactions was enabled by staining the sections using a DAB peroxidase substrate kit (Cat. No. SK-4100; Vector Laboratories). Sections were then counterstained with Haematoxylin and mounted for further evaluation. Slides were scanned with Panoramic 250 Flash II Slide Scanner (3DHISTECH, Budapest, Hungary) and viewed with 3DHistech Panoramic Viewer Software (3DHISTECH, Budapest, Hungary). All corresponding analysis was performed using the NIH ImageJ software (National Institutes of Health, Bethesda, MD) and further confirmed independently by a staff neuropathologist, Dr. K. Aldape. ImageJ is an open-source analytical/processing software developed at the NIH.

### Statistical analysis

All experiments were performed in triplicate with mean and standard error of the mean reported where necessary. Where appropriate, an unpaired 2-tailed Student's t-test was performed for calculation of significance, which was defined as p < 0.05. Kaplan-Meier survival analysis was performed and the survival was statistically compared by the log-rank test. Probability values less than 0.05 were considered significant.

## SUPPLEMENTARY MATERIALS FIGURES AND TABLES









## References

[1] Calin GA, Croce CM (2006). MicroRNA signatures in human cancers. Nature reviews Cancer.

[2] Croce CM, Calin GA (2005). miRNAs, cancer, and stem cell division. Cell.

[3] Lu J, Getz G, Miska EA, Alvarez-Saavedra E, Lamb J, Peck D, Sweet-Cordero A, Ebert BL, Mak RH, Ferrando AA, Downing JR, Jacks T, Horvitz HR, Golub TR (2005). MicroRNA expression profiles classify human cancers. Nature.

[4] Gaur A, Jewell DA, Liang Y, Ridzon D, Moore JH, Chen C, Ambros VR, Israel MA (2007). Characterization of microRNA expression levels and their biological correlates in human cancer cell lines. Cancer research.

[5] Bahubeshi A, Tischkowitz M, Foulkes WD (2011). miRNA processing and human cancer: DICER1 cuts the mustard. Science translational medicine.

[6] Heravi-Moussavi A, Anglesio MS, Cheng SW, Senz J, Yang W, Prentice L, Fejes AP, Chow C, Tone A, Kalloger SE, Hamel N, Roth A, Ha G, Wan AN, Maines-Bandiera S, Salamanca C (2012). Recurrent somatic DICER1 mutations in nonepithelial ovarian cancers. The New England journal of medicine.

[7] Martello G, Rosato A, Ferrari F, Manfrin A, Cordenonsi M, Dupont S, Enzo E, Guzzardo V, Rondina M, Spruce T, Parenti AR, Daidone MG, Bicciato S, Piccolo S (2010). A MicroRNA targeting dicer for metastasis control. Cell.

[8] Melo SA, Ropero S, Moutinho C, Aaltonen LA, Yamamoto H, Calin GA, Rossi S, Fernandez AF, Carneiro F, Oliveira C, Ferreira B, Liu CG, Villanueva A, Capella G, Schwartz S, Shiekhattar R (2009). A TARBP2 mutation in human cancer impairs microRNA processing and DICER1 function. Nature genetics.

[9] Melo SA, Moutinho C, Ropero S, Calin GA, Rossi S, Spizzo R, Fernandez AF, Davalos V, Villanueva A, Montoya G, Yamamoto H, Schwartz S, Esteller M (2010). A genetic defect in exportin-5 traps precursor microRNAs in the nucleus of cancer cells. Cancer cell.

[10] Merritt WM, Lin YG, Han LY, Kamat AA, Spannuth WA, Schmandt R, Urbauer D, Pennacchio LA, Cheng JF, Nick AM, Deavers MT, Mourad-Zeidan A, Wang H, Mueller P, Lenburg ME, Gray JW (2008). Dicer, Drosha, and outcomes in patients with ovarian cancer. The New England journal of medicine.

[11] Bartel DP (2009). MicroRNAs: target recognition and regulatory functions. Cell.

[12] Hill DA, Ivanovich J, Priest JR, Gurnett CA, Dehner LP, Desruisseau D, Jarzembowski JA, Wikenheiser-Brokamp KA, Suarez BK, Whelan AJ, Williams G, Bracamontes D, Messinger Y, Goodfellow PJ (2009). DICER1 mutations in familial pleuropulmonary blastoma. Science.

[13] Rio Frio T, Bahubeshi A, Kanellopoulou C, Hamel N, Niedziela M, Sabbaghian N, Pouchet C, Gilbert L, O'Brien PK, Serfas K, Broderick P, Houlston RS, Lesueur F, Bonora E, Muljo S, Schimke RN (2011). DICER1 mutations in familial multinodular goiter with and without ovarian Sertoli-Leydig cell tumors. Jama.

[14] Kumar MS, Pester RE, Chen CY, Lane K, Chin C, Lu J, Kirsch DG, Golub TR, Jacks T (2009). Dicer1 functions as a haploinsufficient tumor suppressor. Genes & development.

[15] Lambertz I, Nittner D, Mestdagh P, Denecker G, Vandesompele J, Dyer MA, Marine JC (2010). Monoallelic but not biallelic loss of Dicer1 promotes tumorigenesis in vivo. Cell death and differentiation.

[16] Arrate MP, Vincent T, Odvody J, Kar R, Jones SN, Eischen CM (2010). MicroRNA biogenesis is required for Myc-induced B-cell lymphoma development and survival. Cancer research.

[17] Kumar MS, Lu J, Mercer KL, Golub TR, Jacks T (2007). Impaired microRNA processing enhances cellular transformation and tumorigenesis. Nature genetics.

[18] Stupp R, Hegi ME, Mason WP, van den Bent MJ, Taphoorn MJ, Janzer RC, Ludwin SK, Allgeier A, Fisher B, Belanger K, Hau P, Brandes AA, Gijtenbeek J, Marosi C, Vecht CJ, Mokhtari K (2009). Effects of radiotherapy with concomitant and adjuvant temozolomide versus radiotherapy alone on survival in glioblastoma in a randomised phase III study: 5-year analysis of the EORTC-NCIC trial. The Lancet Oncology.

[19] DeSano JT, Xu L (2009). MicroRNA regulation of cancer stem cells and therapeutic implications. The AAPS journal.

[20] Kefas B, Comeau L, Floyd DH, Seleverstov O, Godlewski J, Schmittgen T, Jiang J, diPierro CG, Li Y, Chiocca EA, Lee J, Fine H, Abounader R, Lawler S, Purow B (2009). The neuronal microRNA miR-326 acts in a feedback loop with notch and has therapeutic potential against brain tumors. The Journal of neuroscience.

[21] Shackleton M, Quintana E, Fearon ER, Morrison SJ (2009). Heterogeneity in cancer: cancer stem cells versus clonal evolution. Cell.

[22] Bao S, Wu Q, McLendon RE, Hao Y, Shi Q, Hjelmeland AB, Dewhirst MW, Bigner DD, Rich JN (2006). Glioma stem cells promote radioresistance by preferential activation of the DNA damage response. Nature.

[23] Liu G, Yuan X, Zeng Z, Tunici P, Ng H, Abdulkadir IR, Lu L, Irvin D, Black KL, Yu JS (2006). Analysis of gene expression and chemoresistance of CD133+ cancer stem cells in glioblastoma. Molecular cancer.

[24] Charles N, Ozawa T, Squatrito M, Bleau AM, Brennan CW, Hambardzumyan D, Holland EC (2010). Perivascular nitric oxide activates notch signaling and promotes stem-like character in PDGF-induced glioma cells. Cell stem cell.

[25] Calabrese C, Poppleton H, Kocak M, Hogg TL, Fuller C, Hamner B, Oh EY, Gaber MW, Finklestein D, Allen M, Frank A, Bayazitov IT, Zakharenko SS, Gajjar A, Davidoff A, Gilbertson RJ (2007). A perivascular niche for brain tumor stem cells. Cancer cell.

[26] Hambardzumyan D, Becher OJ, Rosenblum MK, Pandolfi PP, Manova-Todorova K, Holland EC (2008). PI3K pathway regulates survival of cancer stem cells residing in the perivascular niche following radiation in medulloblastoma in vivo. Genes & development.

[27] Folkins C, Shaked Y, Man S, Tang T, Lee CR, Zhu Z, Hoffman RM, Kerbel RS (2009). Glioma tumor stem-like cells promote tumor angiogenesis and vasculogenesis via vascular endothelial growth factor and stromal-derived factor 1. Cancer research.

[28] Gilbertson RJ, Rich JN (2007). Making a tumour's bed: glioblastoma stem cells and the vascular niche. Nature reviews Cancer.

[29] Hjelmeland AB, Lathia JD, Sathornsumetee S, Rich JN (2011). Twisted tango: brain tumor neurovascular interactions. Nature neuroscience.

[30] Singh SK, Hawkins C, Clarke ID, Squire JA, Bayani J, Hide T, Henkelman RM, Cusimano MD, Dirks PB (2004). Identification of human brain tumour initiating cells. Nature.

[31] Ravi A, Gurtan AM, Kumar MS, Bhutkar A, Chin C, Lu V, Lees JA, Jacks T, Sharp PA (2012). Proliferation and tumorigenesis of a murine sarcoma cell line in the absence of DICER1. Cancer cell.

[32] Francia S, Michelini F, Saxena A, Tang D, de Hoon M, Anelli V, Mione M, Carninci P, d'Adda di Fagagna F (2012). Site-specific DICER and DROSHA RNA products control the DNA-damage response. Nature.

[33] Wei W, Ba Z, Gao M, Wu Y, Ma Y, Amiard S, White CI, Rendtlew Danielsen JM, Yang YG, Qi Y (2012). A role for small RNAs in DNA double-strand break repair. Cell.

[34] Wei J, Barr J, Kong LY, Wang Y, Wu A, Sharma AK, Gumin J, Henry V, Colman H, Sawaya R, Lang FF, Heimberger AB (2010). Glioma-associated cancer-initiating cells induce immunosuppression. Clinical cancer research.

[35] Wang WC, Lin FM, Chang WC, Lin KY, Huang HD, Lin NS (2009). miRExpress: analyzing high-throughput sequencing data for profiling microRNA expression. BMC bioinformatics.

[36] Kozomara A, Griffiths-Jones S (2014). miRBase: annotating high confidence microRNAs using deep sequencing data. Nucleic acids research.

[37] van den Beucken T, Koch E, Chu K, Rupaimoole R, Prickaerts P, Adriaens M, Voncken JW, Harris AL, Buffa FM, Haider S, Starmans MH, Yao CQ, Ivan M, Ivan C, Pecot CV, Boutros PC (2014). Hypoxia promotes stem cell phenotypes and poor prognosis through epigenetic regulation of DICER. Nature communications.

[38] Jalali S, Chung C, Foltz W, Burrell K, Singh S, Hill R, Zadeh G (2014). MRI biomarkers identify the differential response of glioblastoma multiforme to anti-angiogenic therapy. Neuro-oncology.

[39] Chung C, Jalali S, Foltz W, Burrell K, Wildgoose P, Lindsay P, Graves C, Camphausen K, Milosevic M, Jaffray D, Zadeh G, Menard C (2013). Imaging biomarker dynamics in an intracranial murine glioma study of radiation and antiangiogenic therapy. International journal of radiation oncology, biology, physics.

[40] Arifin DY, Lee KY, Wang CH (2009). Chemotherapeutic drug transport to brain tumor. Journal of controlled release.

[41] Derderian CA, Wink JD, McGrath JL, Collinsworth A, Bartlett SP, Taylor JA (2015). Volumetric changes in cranial vault expansion: comparison of fronto-orbital advancement and posterior cranial vault distraction osteogenesis. Plastic and reconstructive surgery.

[42] Burrell K, Hill RP, Zadeh G (2012). High-resolution in-vivo analysis of normal brain response to cranial irradiation. PloS one.

[43] Salehi F, Jalali S, Alkins R, Lee JI, Lwu S, Burrell K, Gentili F, Croul S, Zadeh G (2013). Proteins involved in regulating bone invasion in skull base meningiomas. Acta neurochirurgica.

[44] Liao Y, Lonnerdal B (2010). Global microRNA characterization reveals that miR-103 is involved in IGF-1 stimulated mouse intestinal cell proliferation. PloS one.

[45] Shang C, Hong Y, Guo Y, Liu YH, Xue YX (2014). MiR-210 up-regulation inhibits proliferation and induces apoptosis in glioma cells by targeting SIN3A. Medical science monitor.

[46] Huang J, Sun C, Wang S, He Q, Li D (2015). microRNA miR-10b inhibition reduces cell proliferation and promotes apoptosis in non-small cell lung cancer (NSCLC) cells. Molecular bioSystems.

[47] Shang C, Guo Y, Hong Y, Liu YH, Xue YX (2015). MiR-21 up-regulation mediates glioblastoma cancer stem cells apoptosis and proliferation by targeting FASLG. Molecular biology reports.

[48] Mao DD, Gujar AD, Mahlokozera T, Chen I, Pan Y, Luo J, Brost T, Thompson EA, Turski A, Leuthardt EC, Dunn GP, Chicoine MR, Rich KM, Dowling JL, Zipfel GJ, Dacey RG (2015). A CDC20-APC/SOX2 Signaling Axis Regulates Human Glioblastoma Stem-like Cells. Cell reports.

[49] Berezovsky AD, Poisson LM, Cherba D, Webb CP, Transou AD, Lemke NW, Hong X, Hasselbach LA, Irtenkauf SM, Mikkelsen T, deCarvalho AC (2014). Sox2 promotes malignancy in glioblastoma by regulating plasticity and astrocytic differentiation. Neoplasia.

[50] Stoltz K, Sinyuk M, Hale JS, Wu Q, Otvos B, Walker K, Vasanji A, Rich JN, Hjelmeland AB, Lathia JD (2015). Development of a Sox2 reporter system modeling cellular heterogeneity in glioma. Neuro-oncology.

[51] Singh SK, Vartanian A, Burrell K, Zadeh G (2012). A microRNA Link to Glioblastoma Heterogeneity. Cancers.

[52] Lyle S, Hoover K, Colpan C, Zhu Z, Matijasevic Z, Jones SN (2014). Dicer cooperates with p53 to suppress DNA damage and skin carcinogenesis in mice. PloS one.

[53] Wei F, Liu Y, Guo Y, Xiang A, Wang G, Xue X, Lu Z (2013). miR-99b-targeted mTOR induction contributes to irradiation resistance in pancreatic cancer. Molecular cancer.

[54] Bazzini AA, Lee MT, Giraldez AJ (2012). Ribosome profiling shows that miR-430 reduces translation before causing mRNA decay in zebrafish. Science.

[55] Djuranovic S, Nahvi A, Green R (2012). miRNA-mediated gene silencing by translational repression followed by mRNA deadenylation and decay. Science.

[56] Fish JE, Santoro MM, Morton SU, Yu S, Yeh RF, Wythe JD, Ivey KN, Bruneau BG, Stainier DY, Srivastava D (2008). miR-126 regulates angiogenic signaling and vascular integrity. Developmental cell.

[57] Chen J, Li Y, Yu TS, McKay RM, Burns DK, Kernie SG, Parada LF (2012). A restricted cell population propagates glioblastoma growth after chemotherapy. Nature.

[58] Chiosea S, Jelezcova E, Chandran U, Luo J, Mantha G, Sobol RW, Dacic S (2007). Overexpression of Dicer in precursor lesions of lung adenocarcinoma. Cancer research.

[59] Ma Z, Swede H, Cassarino D, Fleming E, Fire A, Dadras SS (2011). Up-regulated Dicer expression in patients with cutaneous melanoma. PloS one.

[60] Slezak-Prochazka I, Durmus S, Kroesen BJ, van den Berg A (2010). MicroRNAs, macrocontrol: regulation of miRNA processing. Rna.

[61] Winter J, Jung S, Keller S, Gregory RI, Diederichs S (2009). Many roads to maturity: microRNA biogenesis pathways and their regulation. Nature cell biology.

[62] Stewart ZA, Leach SD, Pietenpol JA (1999). p21(Waf1/Cip1) inhibition of cyclin E/Cdk2 activity prevents endoreduplication after mitotic spindle disruption. Molecular and cellular biology.

[63] Deng W, Zhou Y, Tiwari AF, Su H, Yang J, Zhu D, Lau VM, Hau PM, Yip YL, Cheung AL, Guan XY, Tsao SW (2015). p21/Cyclin E pathway modulates anticlastogenic function of Bmi-1 in cancer cells. International journal of cancer.

[64] Khoshnaw SM, Rakha EA, Abdel-Fatah TM, Nolan CC, Hodi Z, Macmillan DR, Ellis IO, Green AR (2012). Loss of Dicer expression is associated with breast cancer progression and recurrence. Breast cancer research and treatment.

[65] Stratmann J, Wang CJ, Gnosa S, Wallin A, Hinselwood D, Sun XF, Zhang H (2011). Dicer and miRNA in relation to clinicopathological variables in colorectal cancer patients. BMC cancer.

[66] Rodriguez FJ, Orr BA, Ligon KL, Eberhart CG (2012). Neoplastic cells are a rare component in human glioblastoma microvasculature. Oncotarget.

[67] Cheng L, Huang Z, Zhou W, Wu Q, Donnola S, Liu JK, Fang X, Sloan AE, Mao Y, Lathia JD, Min W, McLendon RE, Rich JN, Bao S (2013). Glioblastoma stem cells generate vascular pericytes to support vessel function and tumor growth. Cell.

[68] Dews M, Homayouni A, Yu D, Murphy D, Sevignani C, Wentzel E, Furth EE, Lee WM, Enders GH, Mendell JT, Thomas-Tikhonenko A (2006). Augmentation of tumor angiogenesis by a Myc-activated microRNA cluster. Nature genetics.

[69] Figliolini F, Cantaluppi V, De Lena M, Beltramo S, Romagnoli R, Salizzoni M, Melzi R, Nano R, Piemonti L, Tetta C, Biancone L, Camussi G (2014). Isolation, characterization and potential role in beta cell-endothelium cross-talk of extracellular vesicles released from human pancreatic islets. PloS one.

[70] Minella AC, Swanger J, Bryant E, Welcker M, Hwang H, Clurman BE (2002). p53 and p21 form an inducible barrier that protects cells against cyclin E-cdk2 deregulation. Current biology: CB.

