# Supplementary material for: DICER governs characteristics of glioma stem cells and the resulting tumors in xenograft mouse models of glioblastoma
Source: Oncotarget. 2016 Jul 13;7(35):56431–46. doi: 10.18632/oncotarget.10570 (PMC5302925; doi:10.18632/oncotarget.10570)
Supplement: Supplementary file 1 [file oncotarget-07-56431-s001.pdf]

DICER governs characteristics of glioma stem cells and the resulting tumors in xenograft mouse models of glioblastoma

SUPPLEMENTARY FIGURES AND TABLES

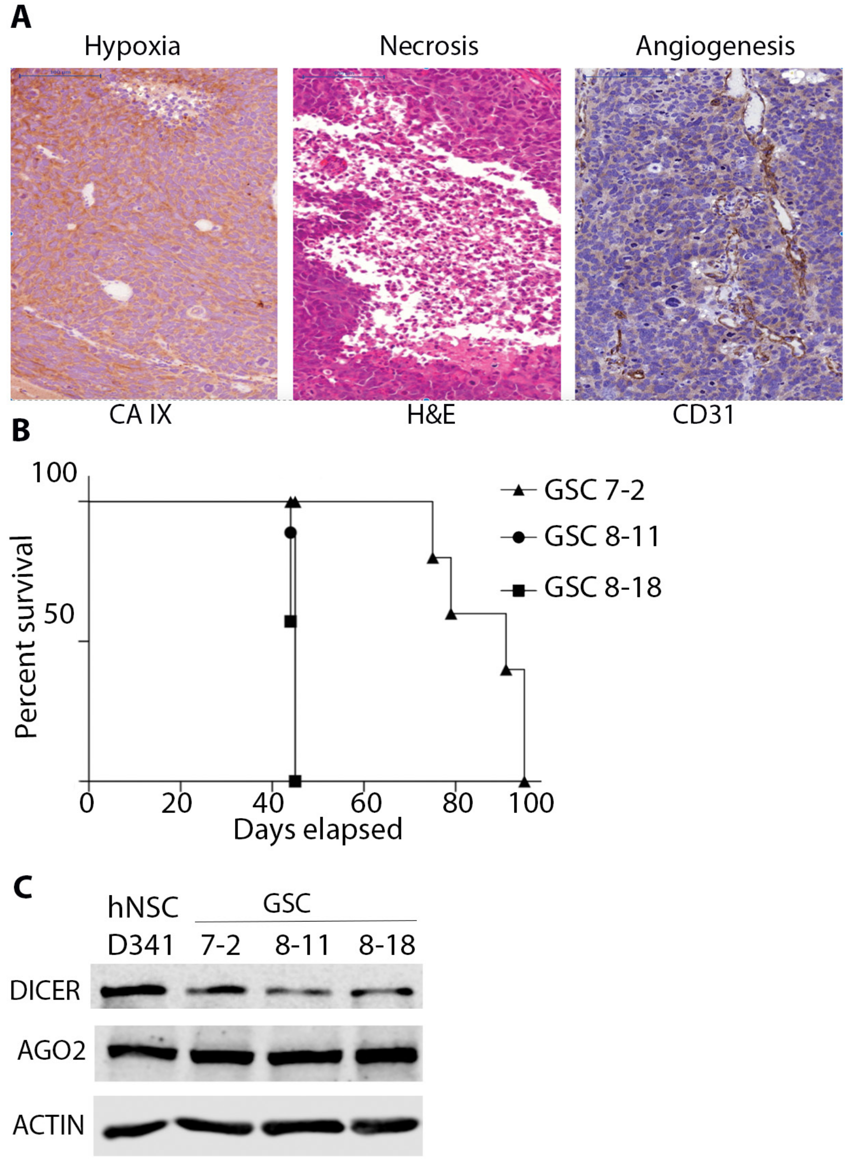

Supplementary Figure S1: Characterization of tumors generated by GSCs in mouse xenografts. GSC 7-2, 8-11, and 8-18 cells (1000 cells) were injected into NOD/SCID mice and moribund mice were sacrificed. **A.** Immunohistochemical analysis of CA IX (hypoxia marker), CD31 (endothelial marker), and H&E staining of tumor section to show necrotic and hypoxic regions as hallmarks of GB tumors. **B.** Kaplan-Meier survival analysis of mice injected with three different GSC lines demonstrating low overall survival in mice bearing these tumors. **C.** Western blot analysis of DICER and AGO2 expression in hNSC D341 cells compared to three GSC lines, showing decreased DICER but no obvious change in AGO2 expression in three GSC lines compared to the NSC line tested.

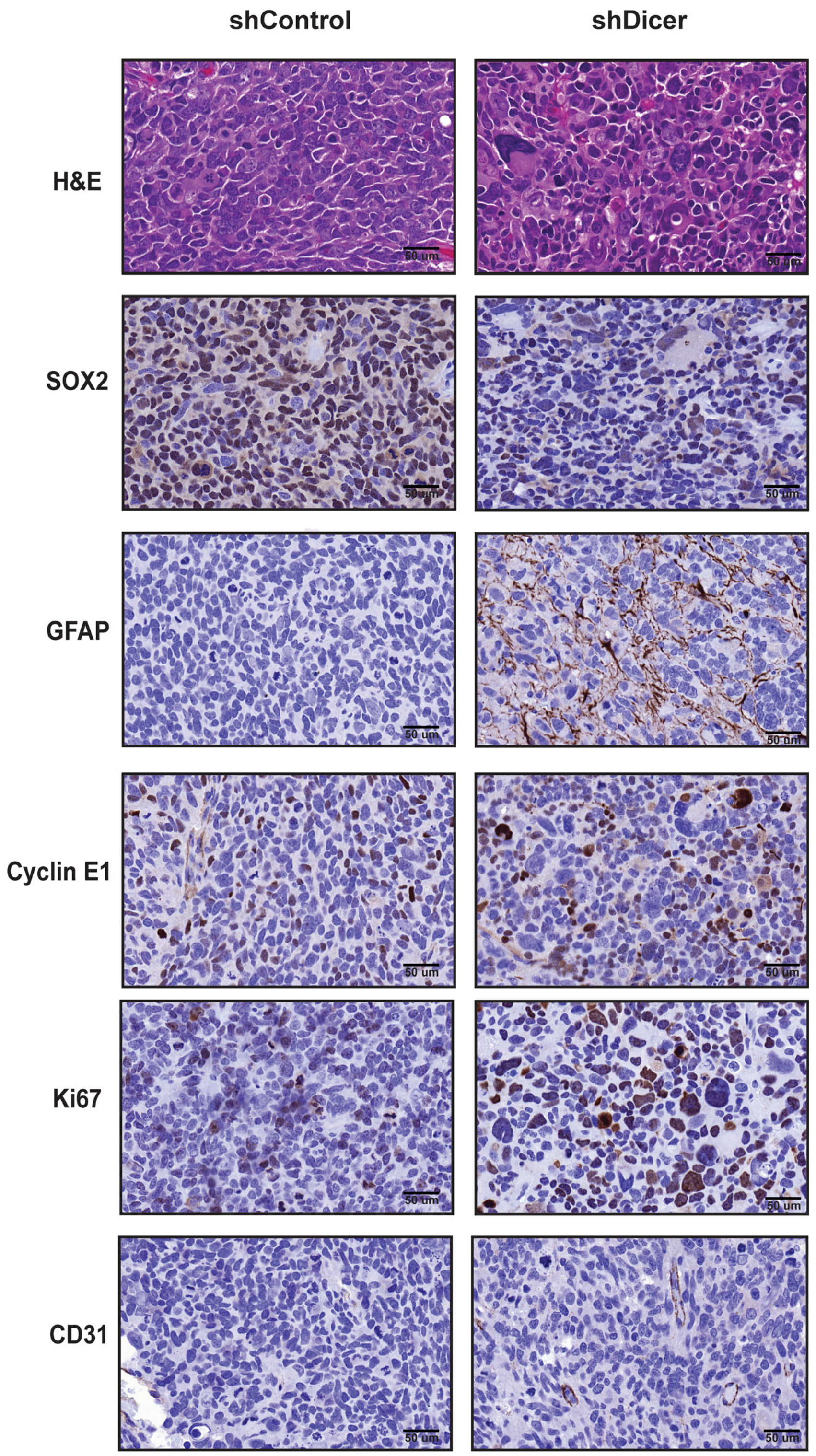

Supplementary Figure S2: Immunohistochemical analysis of stemness, differentiation, and proliferation marker genes in tumors derived from GSC 8-11 cells. IHC analysis of FFPE brain sections collected from mice injected with shDICER or shControl treated GSC 8-11 cells were performed using the indicated antibodies.

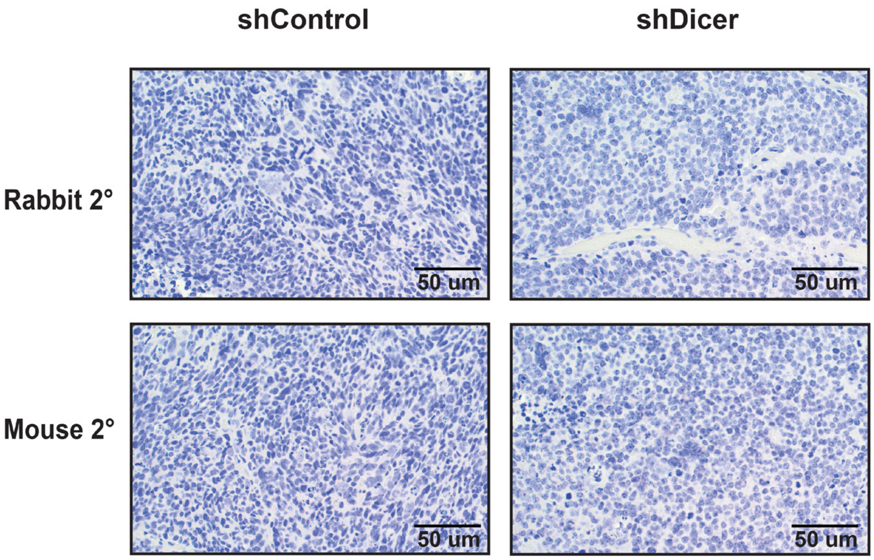

Supplementary Figure S3: Analysis of specificity of antibodies used for IHC. Tumor sections from shControl and shDICER tumors were incubated with secondary antibodies raised in rabbit or mouse (without primary antibody incubation) to determine the degree of their non-specific binding to the tissue sections. The results from these slides were used as negative controls for all other IHC experiments.

Supplementary Table S1: List of forward and reverse primers used for qRT-PCR assays

See Supplementary File 1

Supplementary Table S2: List of antibodies used for Western blotting and IHC

Antibody	*Clone*	*Dilution*	*Source*	*Assay*
CD31	Polyclonal	0.1	Abcam	IHC
SOX2	Monoclonal	0.1	Cell Signaling	IHC
GFAP	Monoclonal	0.3	Dako	IHC
Ki67	Monoclonal	0.4	Abcam	IHC
BMI1	Monoclonal	0.3	Cell Singaling	IHC
OLIG2	Polyclonal	0.4	Millipore	IHC
CCNE	Monoclonal	0.1	Abcam	IHC/WB
p21	Polyclonal	0.2	Santa Cruz	WB
DICER	Polyclonal	0.7	Cell Signaling	WB
AGO2	Polyclonal	0.4	Abcam	WB
ACTIN	Monoclonal	0.7	Cell Singaling	WB

Supplementary Table S3: List of miRNAs specifically bound to RISC complex in GSC 7-2

See Supplementary File 2

Supplementary Table S4: Complete List of miRNAs decreased (>2-fold) in shDICER versus shControl GSC 7-2 cells based on miRNA PCR array results

See Supplementary File 3
