# Supplementary material for: DICER governs characteristics of glioma stem cells and the resulting tumors in xenograft mouse models of glioblastoma
Source: Oncotarget. 2016 Jul 13;7(35):56431–46. doi: 10.18632/oncotarget.10570 (PMC5302925; doi:10.18632/oncotarget.10570)
Supplement: Supplementary file 2 [file oncotarget-07-56431-s002.docx]

**Supplemental Table 1. List of forward and reverse primers used for real-time qPCR assays.**

| **Gene Name** | **Primer Sequence (5'-3')** |
| --- | --- |
| h.qRT-F_DICER1 | TTAACCTTTTGGTGTTTGATGAGTGT |
| h.qRT-R_DICER1 | GCGAGGACATGATGGACAATT |
| h.qRT-F_CDKN1A | TGTCCGTCAGAACCCATGC |
| h.qRT-R_CDKN1A | AAAGTCGAAGTTCCATCGCTC |
| h.qRT-F_CDKN1B | ATCACAAACCCCTAGAGGGCA |
| h.qRT-R_CDKN1B | GGGTCTGTAGTAGAACTCGGG |
| h.qRT-F_CDX2 | GACGTGAGCATGTACCCTAGC |
| h.qRT-R_CDX2 | GCGTAGCCATTCCAGTCCT |
| h.qRT-F_CREB1 | TTAACCATGACCAATGCAGCA |
| h.qRT-R_CREB1 | TGGTATGTTTGTACGTCTCCAGA |
| h.qRT-F_CSF1 | TGGCGAGCAGGAGTATCAC |
| h.qRT-R_CSF1 | AGGTCTCCATCTGACTGTCAAT |
| h.qRT-F_HMOX1 | AAGACTGCGTTCCTGCTCAAC |
| h.qRT-R_HMOX1 | AAAGCCCTACAGCAACTGTCG |
| h.qRT-F_IL8 | ACTGAGAGTGATTGAGAGTGGAC |
| h.qRT-R_IL8 | AACCCTCTGCACCCAGTTTTC |
| h.qRT-F_MET | AGCGTCAACAGAGGGACCT |
| h.qRT-R_MET | GCAGTGAACCTCCGACTGTATG |
| h.qRT-F_MMP9 | TGTACCGCTATGGTTACACTCG |
| h.qRT-R_MMP9 | GGCAGGGACAGTTGCTTCT |
| h.qRT-F_MTOR | TCCGAGAGATGAGTCAAGAGG |
| h.qRT-R_MTOR | CACCTTCCACTCCTATGAGGC |
| h.qRT-F_NOTCH1 | GAGGCGTGGCAGACTATGC |
| h.qRT-R_NOTCH1 | CTTGTACTCCGTCAGCGTGA |
| h.qRT-F_SIRT1 | TGTGTCATAGGTTAGGTGGTGA |
| h.qRT-R_SIRT1 | AGCCAATTCTTTTTGTGTTCGTG |
| h.qRT-F_SOX2 | GCTCTTGGCTCCATGGGTTC |
| h.qRT-R_SOX2 | GCTGATCATGTCCCGGAGGT |
| h.qRT-F_BMI1 | CTGCTCAACATCAGGTCAGATA |
| h.qRT-R_BMI1 | CACTTCTCCCCTGTCTTCATTA |
| h.qRT-F_GFAP | CAAGATGAAACCAACCTGAGGCT |
| h.qRT-R_GFAP | GGCTTGGCCACATCCATCT |
| h.qRT-F_CCNE1 | GAGGAAGGCAAACGTGACC |
| h.qRT-R_CCNE1 | TGTCCCAAGGCTGGCTCC |
| h.qRT-F_NESTIN | CTGAGGCCTCTCTTCTTCCA |
| h.qRT-R_NESTIN | ACTCCTGTACCGGGTCTCCT |
| h.qRT-F_CD31 | ATTGCAGTGGTTATCATCGGAGTG |
| h.qRT-R_CD31 | CTCGTTGTTGGAGTTCAGAAGTGG |
| h.qRT-F_ACTIN | TGCGTGACATCAAAGAGAAG |
| h.qRT-R_ACTIN | GATGCCACAGGATTCCATA |
| h.qRT-F_GAPDH | GAAGGTGAAGGTCGGAGT |
| h.qRT-R_GAPDH | CATGGGTGGAATCATATTGGA |
| h.qRT-F_EFNA3 | CATGCGGTGTACTGGAACAG |
| h.qRT-R_EFNA3 | AGATAGTCGTTCACGTTCACCT |
| h.qRT-F_CASP8AP2 | ACACAGTCGAGTAGACTCTCAAA |
| h.qRT-R_CASP8AP2 | AGGAAGTGATGCTCGTTCAGA |
| h.qRT-F_VMP1 | TTTCCCGAACCACCCTATCCT |
| h.qRT-R_VMP1 | GATTGCTGTACCGATACCCCA |
| h.qRT-F_MNT | TCGGAACCAGAGAAGTCCAC |
| h.qRT-R_MNT | CGCTCCATTTCATGCTCATA |
| h.qRT-F_SRSF1 | CCGCAGGGAACAACGATTG |
| h.qRT-R_SRSF1 | GCCGTATTTGTAGAACACGTCCT |
| h.qRT-F_HOXD10 | CAGCAGCGCCAGCATGT |
| h.qRT-R_HOXD10 | TTCACTTCTCTTTTGGCCAGAGA |
| h.qRT-F_KLF4 | GCCTTTGCTAACACTGATGA |
| h.qRT-R_KLF4 | GATGGGCAAGTTCGTGTT |
| h.qRT-F_TRA2B | TAGGCGTTCAAGAGGATTTG |
| h.qRT-R_TRA2B | TTCCATTGGCACGTTCTTTA |
| h.qRT-F_FBXW11 | GGAACATCATCTGTGATCGTCTC |
| h.qRT-R_FBXW11 | TGGTAAAGCGGTAATAAAGTCCC |
| h.qRT-F_SERBP1 | TAGACCGATTATTGACCGACCT |
| h.qRT-R_SERBP1 | GTTTGCCACGAGAATCAAATCC |
| h.qRT-F_CREB1 | TTAACCATGACCAATGCAGCA |
| h.qRT-R_CREB1 | TGGTATGTTTGTACGTCTCCAGA |
| h.qRT-F_CCNE1 | CTCCAGGAAGAGGAAGGCAA |
| h.qRT-R_CCNE1 | TCGATTTTGGCCATTTCTTCA |
| h.qRT-F_CDK2 | GCTAGCAGACTTTGGACTAGCCAG |
| h.qRT-R_CDK2 | AGCTCGGTACCACAGGGTCA |
| h.qRT-F_SPRY2 | CCTACTGTCGTCCCAAGACCT |
| h.qRT-R_SPRY2 | GGGGCTCGTGCAGAAGAAT |
