# Supplementary material for: DICER governs characteristics of glioma stem cells and the resulting tumors in xenograft mouse models of glioblastoma
Source: Oncotarget. 2016 Jul 13;7(35):56431–46. doi: 10.18632/oncotarget.10570 (PMC5302925; doi:10.18632/oncotarget.10570)
Supplement: Supplementary file 3 [file oncotarget-07-56431-s003.docx]

**Supplemental Table 3. List of miRNAs enriched in RISC complex in GSC 7-2.**

| **MicroRNA** | **Relative Levels** | **Binding Rel. to IgG** |
| --- | --- | --- |
| hsa-miR-96-5p | 0.05 | 143.01 |
| hsa-miR-29b-3p | 0.03 | 97.68 |
| hsa-miR-216a-5p | 0.23 | 89.88 |
| hsa-miR-193b-3p | 0.01 | 71.51 |
| hsa-miR-15a-5p | 0.08 | 69.55 |
| hsa-miR-27a-3p | 0.07 | 67.65 |
| hsa-miR-22-3p | 0.05 | 64.45 |
| hsa-miR-135b-5p | 0.11 | 57.68 |
| hsa-miR-101-3p | 0.14 | 53.45 |
| hsa-miR-551b-3p | 0.07 | 52.71 |
| hsa-miR-124-3p | 0.06 | 42.81 |
| hsa-miR-197-3p | 0.04 | 39.67 |
| hsa-miR-301a-3p | 0.13 | 33.13 |
| hsa-miR-99a-3p | 0.00 | 33.13 |
| hsa-miR-340-5p | 0.03 | 27.47 |
| hsa-miR-29c-3p | 0.47 | 27.10 |
| hsa-miR-210 | 0.02 | 27.10 |
| hsa-miR-140-3p | 0.06 | 23.92 |
| hsa-miR-19a-3p | 2.28 | 23.10 |
| hsa-miR-425-3p | 0.01 | 23.10 |
| hsa-miR-181c-5p | 0.14 | 22.16 |
| hsa-miR-345-5p | 0.06 | 21.56 |
| hsa-miR-23a-3p | 0.23 | 20.82 |
| hsa-miR-181a-5p | 0.16 | 20.11 |
| hsa-miR-378a-5p | 0.01 | 19.97 |
| hsa-miR-19b-3p | 1.74 | 19.56 |
| hsa-miR-339-3p | 0.02 | 19.56 |
| hsa-miR-153 | 0.02 | 19.43 |
| hsa-miR-31-5p | 0.02 | 19.43 |
| hsa-miR-132-3p | 0.03 | 19.29 |
| hsa-miR-30e-5p | 1.05 | 19.03 |
| hsa-miR-106b-5p | 2.38 | 18.90 |
| hsa-miR-130b-3p | 0.16 | 17.75 |
| hsa-miR-107 | 0.04 | 17.63 |
| hsa-miR-340-3p | 0.01 | 17.39 |
| hsa-miR-324-3p | 0.03 | 17.27 |
| hsa-miR-29a-3p | 0.38 | 16.80 |
| hsa-miR-186-5p | 0.48 | 16.68 |
| hsa-miR-138-5p | 0.01 | 16.68 |
| hsa-let-7i-5p | 0.23 | 16.34 |
| hsa-miR-21-5p | 0.48 | 16.22 |
| hsa-miR-424-5p | 0.45 | 16.22 |
| hsa-miR-30b-5p | 0.42 | 15.14 |
| hsa-let-7f-5p | 0.13 | 15.03 |
| hsa-miR-99b-5p | 0.33 | 14.93 |
| hsa-miR-103a-3p | 0.77 | 14.83 |
| hsa-miR-16-5p | 4.14 | 14.72 |
| hsa-miR-324-5p | 0.05 | 14.72 |
| hsa-miR-574-3p | 0.01 | 14.72 |
| hsa-miR-423-3p | 0.20 | 14.03 |
| hsa-miR-24-3p | 0.67 | 13.83 |
| hsa-miR-30a-5p | 1.11 | 13.74 |
| hsa-miR-100-5p | 0.03 | 13.74 |
| hsa-miR-148b-3p | 0.20 | 13.36 |
| hsa-miR-320a | 0.31 | 13.18 |
| hsa-miR-128 | 0.14 | 13.18 |
| hsa-miR-15b-3p | 0.10 | 13.00 |
| hsa-miR-532-5p | 0.03 | 13.00 |
| hsa-let-7d-5p | 0.16 | 12.91 |
| hsa-miR-148a-3p | 0.75 | 12.82 |
| hsa-miR-449a | 0.01 | 12.64 |
| hsa-miR-135a-5p | 1.20 | 12.30 |
| hsa-miR-27b-3p | 0.36 | 12.30 |
| hsa-miR-106b-3p | 0.05 | 12.13 |
| hsa-miR-20a-5p | 7.41 | 12.04 |
| hsa-miR-486-5p | 0.06 | 11.79 |
| hsa-miR-183-3p | 0.02 | 11.79 |
| hsa-miR-342-3p | 0.27 | 11.55 |
| hsa-miR-26b-5p | 0.20 | 11.39 |
| hsa-miR-221-3p | 0.31 | 11.31 |
| hsa-miR-374a-5p | 0.29 | 11.16 |
| hsa-miR-192-5p | 0.02 | 11.16 |
| hsa-miR-185-5p | 0.05 | 11.00 |
| hsa-miR-182-5p | 0.65 | 10.78 |
| hsa-miR-99a-5p | 2.46 | 10.70 |
| hsa-miR-29a-5p | 0.02 | 10.70 |
| hsa-miR-320b | 0.01 | 10.70 |
| hsa-miR-93-5p | 4.79 | 10.63 |
| hsa-let-7g-5p | 1.42 | 10.56 |
| hsa-miR-28-5p | 0.08 | 10.56 |
| hsa-miR-190a | 0.02 | 10.34 |
| hsa-miR-17-5p | 4.59 | 10.20 |
| hsa-miR-15b-5p | 3.34 | 10.20 |
| hsa-miR-26a-5p | 2.25 | 10.13 |
| hsa-miR-18a-5p | 0.95 | 9.99 |
| hsa-let-7b-5p | 0.36 | 9.71 |
| hsa-miR-484 | 0.26 | 9.71 |
| hsa-miR-125a-5p | 1.08 | 9.65 |
| hsa-miR-30d-5p | 0.81 | 9.65 |
| hsa-miR-146b-5p | 0.02 | 9.58 |
| hsa-miR-25-3p | 2.50 | 9.51 |
| hsa-miR-30c-5p | 2.01 | 9.51 |
| hsa-miR-194-5p | 0.04 | 9.51 |
| hsa-miR-152 | 0.04 | 9.25 |
| hsa-miR-98-5p | 0.06 | 9.19 |
| hsa-miR-140-5p | 0.05 | 9.06 |
| hsa-miR-425-5p | 0.29 | 8.94 |
| hsa-miR-18a-3p | 0.05 | 8.94 |
| hsa-miR-92b-3p | 0.02 | 8.94 |
| hsa-miR-183-5p | 0.20 | 8.88 |
| hsa-miR-92a-3p | 2.41 | 8.63 |
| hsa-miR-92a-1-5p | 0.01 | 8.51 |
| hsa-miR-365b-3p | 0.13 | 8.40 |
| hsa-miR-423-5p | 0.13 | 8.40 |
| hsa-miR-491-5p | 0.00 | 8.28 |
| hsa-miR-885-5p | 0.01 | 8.22 |
| hsa-miR-421 | 0.11 | 7.84 |
| hsa-miR-204-5p | 0.08 | 7.67 |
| hsa-miR-500a-5p | 0.03 | 7.62 |
| hsa-miR-10b-5p | 0.01 | 7.62 |
| hsa-miR-22-5p | 0.01 | 7.57 |
| hsa-miR-217 | 1.78 | 7.46 |
| hsa-miR-195-5p | 1.27 | 7.46 |
| hsa-miR-95 | 0.02 | 7.31 |
| hsa-miR-196b-5p | 0.19 | 7.16 |
| hsa-miR-181d | 0.02 | 7.16 |
| hsa-miR-20a-3p | 0.17 | 7.01 |
| hsa-miR-652-3p | 0.05 | 7.01 |
| hsa-miR-542-3p | 0.07 | 6.82 |
| hsa-miR-200b-3p | 0.01 | 6.82 |
| hsa-miR-129-5p | 0.01 | 6.59 |
| hsa-miR-9-5p | 7.01 | 6.41 |
| hsa-miR-23b-3p | 0.80 | 6.41 |
| hsa-miR-378a-3p | 0.29 | 6.23 |
| hsa-let-7e-5p | 0.18 | 6.23 |
| hsa-miR-151a-5p | 0.54 | 6.19 |
| hsa-miR-20b-5p | 1.49 | 6.15 |
| hsa-let-7c | 0.73 | 6.02 |
| hsa-miR-28-3p | 0.09 | 6.02 |
| hsa-miR-129-1-3p | 0.08 | 5.98 |
| hsa-miR-505-3p | 0.10 | 5.86 |
| hsa-miR-151a-3p | 0.23 | 5.78 |
| hsa-miR-93-3p | 0.12 | 5.78 |
| hsa-miR-361-5p | 0.07 | 5.58 |
| hsa-let-7a-5p | 1.48 | 5.54 |
| hsa-miR-191-3p | 0.00 | 5.43 |
| hsa-miR-454-3p | 0.30 | 5.10 |
| hsa-miR-708-5p | 0.07 | 5.03 |
| hsa-miR-129-2-3p | 0.04 | 5.03 |
| hsa-miR-374c-5p | 0.53 | 4.99 |
| hsa-miR-424-3p | 0.01 | 4.89 |
| hsa-miR-125b-5p | 2.83 | 4.56 |
| hsa-miR-191-5p | 2.46 | 4.32 |
| hsa-miR-181b-5p | 0.40 | 4.20 |
| hsa-miR-27b-5p | 0.02 | 3.71 |
| hsa-miR-744-5p | 0.15 | 3.39 |
| hsa-miR-32-3p | 0.01 | 3.25 |
| hsa-miR-9-3p | 1.37 | 3.20 |
| hsa-miR-30e-3p | 0.16 | 3.14 |
