# Supplementary material for: DICER governs characteristics of glioma stem cells and the resulting tumors in xenograft mouse models of glioblastoma
Source: Oncotarget. 2016 Jul 13;7(35):56431–46. doi: 10.18632/oncotarget.10570 (PMC5302925; doi:10.18632/oncotarget.10570)
Supplement: Supplementary file 4 [file oncotarget-07-56431-s004.docx]

**Supplemental Table 4: Complete List of miRNAs decreased (>2-fold) in *DICER* knockdown versus control GSC 7-2 cells based on miRNA PCR array results.**

| **miRNA** | **Fold Change** |
| --- | --- |
| hsa-let-7b-5p | -18.1 |
| hsa-miR-98-5p | -14.0 |
| hsa-let-7i-5p | -11.8 |
| hsa-let-7d-5p | -9.2 |
| hsa-let-7g-5p | -7.3 |
| hsa-miR-301a-3p | -6.2 |
| hsa-miR-129-2-3p | -5.4 |
| hsa-let-7f-5p | -5.4 |
| hsa-miR-99b-5p | -4.8 |
| hsa-let-7c | -4.5 |
| hsa-miR-652-3p | -4.3 |
| hsa-miR-107 | -4.2 |
| hsa-miR-210 | -4.0 |
| hsa-miR-330-3p | -3.9 |
| hsa-miR-105-5p | -3.8 |
| hsa-miR-342-5p | -3.7 |
| hsa-let-7e-5p | -3.6 |
| hsa-miR-103a-3p | -3.5 |
| hsa-miR-345-5p | -3.5 |
| hsa-miR-484 | -3.5 |
| hsa-miR-181c-5p | -3.4 |
| hsa-miR-129-1-3p | -3.3 |
| hsa-miR-93-5p | -3.1 |
| hsa-miR-200c-3p | -3.1 |
| hsa-miR-421 | -3.0 |
| hsa-miR-146b-5p | -3.0 |
| hsa-miR-324-5p | -3.0 |
| hsa-miR-542-3p | -3.0 |
| hsa-miR-212-3p | -2.9 |
| hsa-miR-29b-2-5p | -2.9 |
| hsa-miR-542-5p | -2.9 |
| hsa-miR-129-5p | -2.8 |
| hsa-miR-324-3p | -2.8 |
| hsa-miR-181a-5p | -2.7 |
| hsa-miR-363-3p | -2.7 |
| hsa-miR-32-3p | -2.7 |
| hsa-miR-425-5p | -2.6 |
| hsa-miR-183-5p | -2.6 |
| hsa-miR-320b | -2.6 |
| hsa-miR-18a-5p | -2.6 |
| hsa-miR-93-3p | -2.6 |
| hsa-miR-196b-5p | -2.5 |
| hsa-miR-222-3p | -2.5 |
| hsa-miR-1180 | -2.5 |
| hsa-miR-18b-5p | -2.5 |
| hsa-miR-454-3p | -2.5 |
| hsa-miR-181b-5p | -2.3 |
| hsa-miR-106b-3p | -2.2 |
| hsa-miR-27a-3p | -2.1 |
| hsa-miR-185-5p | -2.1 |
| hsa-miR-30e-5p | -2.1 |
| hsa-miR-423-5p | -2.0 |
